# Perceived vulnerability to disease in pregnancy and parenthood and its impact on newborn health

**DOI:** 10.1038/s41598-024-71870-w

**Published:** 2024-09-08

**Authors:** Agnieszka Sorokowska, Aleksandra Pytlinska, Tomasz Frackowiak, Piotr Sorokowski, Anna Oleszkiewicz, Michal Mikolaj Stefanczyk, Marta Rokosz

**Affiliations:** 1 Institute of Psychology, University of Wroclaw, ul. Dawida 1, 50-527 Wroclaw, Poland; 2 Smell and Taste Clinic, Dept. of Otorhinolaryngology, Technische Universität Dresden, Fetscherstr. 74, 01307 Dresden, Germany

**Keywords:** Perceived vulnerability to disease (PVD), Pregnancy, Parenthood, Health, Psychology, Health care

## Abstract

Susceptibility to diseases and fear of infections might vary intra-individually, depending on life circumstances. The main aims of the current research were to examine whether perceived vulnerability to disease (PVD) is higher in expectant women and their partners as compared to their non-pregnant peers (Study 1), and to test whether a mother’s disease aversion during pregnancy relates to health of her newborn (Study 2). In Study 1 we collected cross-sectional data from 412 men and women varying in parenthood status. Pregnant female participants were more likely to exhibit higher levels of PVD as compared with childless peers, although mothers also reported relatively high PVD scores. PVD in men, generally lower than that of women, seemed to be rather independent of their parenthood status. In Study 2, a sample of 200 pregnant women completed the PVD scale during the second pregnancy trimester and a follow-up survey after their child was born. We found that PVD in pregnant women was not related to further health outcomes in their newborns. Birth weight, average Apgar score, and general health of a newborn were not associated with the pregnancy-period mother’s PVD score. However, the probability of giving birth to a child with 10 Apgar points was higher in younger mothers and tended to decrease with the increasing number of health issues before pregnancy. Overall, this research contributes to understanding of the health-oriented beliefs of expectant parents and parents of infants, but it also shows that the possible, PVD-related disease avoidance has a relatively little effect on basic markers of a newborn’s health.

## Introduction

Susceptibility to diseases and fear of infections might vary intra-individually, depending on life circumstances. For example, pregnancy is a time of particular vulnerability for a woman. The fetus is a half-foreign element of the female body, and to protect it from maternal immune response, the whole immune system slows down, leaving a woman more prone to diseases^1–3^. However, some researchers point out that the mechanism of the mother's immune system is more complex, and that pregnancy is not a period of immune suppression, but of dynamic immunomodulation that both protects against pathogens and prevents fetal rejection by the maternal immune system^4^. Nevertheless, pregnancy is a difficult time for future parents health-wise, as virtually all infections can result in complications in fetal development. Viral infections that cross the placental barrier and reach the fetus can adversely influence a fetus^5^. Viruses such as HIV, hepatitis A-C, or rubella, even influenza or herpes simplex may predispose the pregnancy to spontaneous abortion, preterm labor, and birth defects such as neurological deficits, blindness, hearing loss, or psychiatric disorders^3,5,6^.

Interestingly, these vulnerable stages of human development are accompanied by some cognitive and behavioral adjustments, potentially decreasing the probability of serious pregnancy complications. Pregnancy relates to increased prophylactic behaviors that can protect women and their children from diseases (e.g.,^1,7–11^). For example, compared to non-pregnant women, pregnant participants show a stronger preference for faces perceived as healthy^9^. Further, longitudinal studies across pregnancy show that in the first trimester, pregnant women exhibit particularly high disgust sensitivity^10,11^. The assumption of increased prophylactic behaviors is also supported by the increased social distance from individuals with symptoms of an infection^12^, or a significant correlation between indicators of immune system activity in pregnant women and their disgust sensitivity^13^. Overall, it can be hypothesized that some female behaviors and preferences are meant to compensate for lower reactivity of the maternal immune system during pregnancy, and to reduce the risk of diseases affecting the unborn child's development. However, it is not entirely clear whether these prophylactic behaviors help reduce a newborn's health risks.

Another critical developmental moment in terms of prophylactic behaviors and disease avoidance is childbirth and young infancy. Parents of small children are expected to be focused on their health and create a protective environment given the high vulnerability of newborns and infants to diseases^14,15^. For many infections, such as smallpox, clinical severity is relatively high in infancy, and a peak immune function is reached around 5–14 years of age^16^ since a child’s immune system continues to develop^15^. Consequently, it may be assumed that health-oriented behaviors and attitudes would likely be pronounced among parents of young children, but this hypothesis has rarely been tested empirically. There are, however, a few studies concerning disgust-related behaviors of parents which support this notion. Mothers of young children (up to 7 years of age) demonstrate a more marked avoidance of disgust elicitors than parents of older children, suggesting a parent–child transmission of disgust-driven behaviors^17^, in line with a reasoning that proper disgust reactions are obtained through social learning^18^. However, contrary to pregnant women, mothers tend to be less disgust sensitive than childless women^19^. This may result from desensitization, i.e., a gradually acquired indifference to various disgust elicitors commonly encountered during childcare (e.g., diseases, exposure to bodily fluids of the offspring, etc.). This phenomenon may be explained by the source effect, as previous studies showed that familiar disgust elicitors are considered as less disgusting than unfamiliar ones^20^. Given that many essential childcare activities are (and historically predominantly have been) performed rather by mothers than fathers (e.g.,^21^), it could be expected that changes in sensitivity to pathogens during pregnancy and the first years of childrearing should be evident particularly (if not only) among women/mothers, and not men/fathers. There are theoretical works that support this potential discrepancy. For instance, Al-Shawaf et al.^22^ state that disease avoidance-related psychological changes apply primarily to women, as “mothers matter more” for an offspring survival. Similarly, considering the childrearing social role of predominantly mothers and offspring’s vulnerability to infections, it is women/mothers who “should be disgusted enough for two”^23^. Consequently, any pregnancy- and childrearing-related changes in vulnerability to disease cues may be found only in women. Notably, a recent study that included also men/fathers showed lowered levels of disgust sensitivity among parents in general^24^, suggesting that parental status may be influential for both women’s and men’s psychology. As such, we expected to see an influence of parental status on perceived vulnerability to disease in mothers, and either a lack of it or a similar effect in men. Additionally, in line with the previous literature (e.g.,^25^), we expected women to declare overall higher levels of vulnerability to disease than men.

Overall, knowledge about health-oriented behaviors and beliefs of people expecting and having children is rather scarce. In the case of parents of small children, studies also tend to focus on beliefs concerning the child, such as parental perception of a child's vulnerability (PPCV; e.g.,^26^), and not on parents’ perception of their own vulnerability to disease. Additionally, previous research mostly fails to examine whether the increased protective behaviors and beliefs that one is vulnerable are observed also among fathers (or partners of pregnant women). Although the magnitude of this effect may be lower than in the case of women, it seems quite logical to expect that it exists; mechanisms promoting disease avoidance could stretch to a man, given that he or his physical/social environment might be a source of potential infections and/or that he needs to take care of his family (i.e., stay healthy). Further, the consequences of disease avoidance during pregnancy have not been examined in the context of further health outcomes in newborn children of mothers varying in PVD levels.

Here, we aimed to examine whether perceived vulnerability to disease is higher in expectant women and their partners as compared to their non-pregnant peers. We hypothesized that the observed effects would be particularly salient among female participants expecting or having a child. Further, in a second study, we explored whether a mother’s disease aversion during pregnancy relates to the health indices of her newborn. Our research included also other variables that seemed potentially important in the context of our predictions. For example, pregnancy and parenthood may generate particularly high stress^27^, which may be even more pronounced in unfavorable socio-economic conditions^28^. Exposure to maternal psychological stress during fetal development has been linked to long-term infant health and development consequences^29,30^, including neurodevelopmental and respiratory issues, and preterm birth^30,31^. In this context, in addition to our main research questions, we decided to investigate the association of stress with parenthood status, and the predictive value of stress and material situation for pregnancy outcomes.

## Study 1

### Materials and methods

#### Participants

In Study 1 we collected cross-sectional data from a sample of 412 childless individuals, expectant parents, and parents from Poland. The sample comprised 138 participants who declared they (or their partner) were not pregnant at the time of the study (86 women and 52 men; *M*_age_ = 26.8 ± 3.03; *M*_SES_ = 4.54 ± 1.20), 117 who expected a child (76 women and 41 men; *M*_age_ = 29.4 ± 2.97; *M*_SES_ = 4.91 ± 1.06), and 157 who had at least one child under two years of age (94 women and 63 men; *M*_age_ = 30.5 ± 3.38; *M*_SES_ = 5.08 ± 1.07). All participants declared being involved in a romantic relationship at the time of the study. The participants provided informed written consent to be included in the study.

#### Methods

The participants completed the Perceived Vulnerability to Disease scale (PVD)^25^, the self-assessment Perceived Stress Scale (PSS-10)^32^, and a brief demographic survey that included questions on age, age of the partner, gender, parenthood status, relationship status and duration, hormonal problems, number of years of completed education (*M* = 17.1 ± 2.39 in our sample) and self-assessed socio-economic status (SES; rated from 1 indicating much lower than the average in my country to 7, meaning much higher than the average in my country).

##### PVD assessment

The 15-item PVD scale^25^ comprises two subscales: (1) Germ Aversion, that focuses on emotional discomfort in contexts associated with high pathogen transmission (e.g., *I prefer to wash my hands pretty soon after shaking someone’s hand*), and (2) Perceived Infectability which covers one’s beliefs about own susceptibility to infectious diseases (e.g., *In general, I am very susceptible to colds, flu and other infectious diseases*). Subscale scores are computed as a total sum or mean score of all items belonging to the subscale. After reverse-scoring of indicated items the higher the score, the higher the perceived vulnerability to disease. The scale shows good convergent and discriminant validity, as the subscales correlate positively with health-related variables such as disgust sensitivity and general hypochondriacal fears and beliefs, but negatively with sociosexual orientation^25^. It also reflects gender, national, and cultural differences that align with existing research on disgust sensitivity, and the geographical differences in prevalence of infectious diseases. Similar to the original study^25^, our data showed an acceptable level of internal consistency for the full scale (Cronbach’s alpha = 0.82 in both our and the original study), the Perceived Infectability subscale, Cronbach’s alpha = 0.90 (0.87 in the original study), and a lower reliability for the Germ Aversion subscale, Cronbach’s alpha = 0.65 (0.74 in the original study). The factors were modestly correlated with each other across the whole sample, *r* = 0.32, *p* < 0.001.

##### Stress assessment

The PSS-10^32^ is a popular tool for measuring psychological stress (exemplary item: *In the last month, how often have you been upset because of something that happened unexpectedly?*). Participants report the frequency of certain psychological and behavioral markers of stress in a past month using a Likert-type 1–5 response format. The scale displays good temporal stability (test–retest correlation 0.85 in the original study), as well as validity, shown by a positive correlation with number and impact of stressful life events^32^. The scale had a satisfactory level of internal consistency (Cronbach’s alpha = 0.88; 0.84-0.86 in the original study).

#### Procedure

Participants were invited to complete a short online survey regarding health-oriented behaviors. The invitation to participate in the study was distributed by the experimenters through the institutional website and social media, the authors’ social media, through invitations sent directly to groups of expectant mothers and young parents and by snowball technique (all participants were invited to resend the link to their friends and acquaintances). We noted in the survey inclusion criteria that we only invite people who are childless, expect their first child, or have one child below two years of age to complete the questionnaires. Not meeting any of these criteria (as indicated in the survey) redirected the participants to a screen where they were thanked for their willingness to complete the study. The participants provided their informed consent to be included in the study and the procedure was approved by the Ethical Committee of the Institute of Psychology, University of Wroclaw. All data are available online^33^. The participants received no compensation for their participation in the study.

#### Data analysis

Statistical analyses were performed with *jamovi* 2.0 statistical package^34^ with the level of significance set to α = 0.05. We computed two separate ANCOVA analyses with Infectability and Germ Aversion PVD subscales as dependent variables, gender (male/female), and parenthood status (no children/pregnant/parent) and their interactions as between-subject factors and age included as a covariate. We also performed two supplementary analyses concerning stress level: an analogous, supplementary ANCOVA with the stress level of our participants as a dependent variable and correlations of perceived vulnerability to disease and stress level (Pearson’s *r* correlations computed for the entire sample and separately for men and women; see Study 1 Supplementary Materials for the full results).

### Results

For Infectability, the interaction effect of gender and parenthood status was non-significant and so was the main parenthood status effect, while the main effect of gender was significant, *F*(1,405) = 5.16, *p* = 0.024, η^2^ = 0.01. Women perceived their infectability as slightly higher than men (*M* = 3.24; 95%CI 3.09–3.40 vs. *M* = 2.95; 95%CI 2.74–3.15). For Germ Aversion, the overall gender*parenthood status interaction effect was non-significant, but again this PVD subscale score was significantly affected by gender, *F*(1,405) = 7.95, *p* = 0.005, η^2^ = 0.02, with women assessing their germ aversion as slightly higher than men (*M* = 3.45; 95%CI 3.38–3.61 vs. *M* = 3.22; 95%CI 3.06–3.37). Germ aversion also tended to be affected by parenthood status, *F*(2,405) = 2.25, *p* = 0.107, and provided the focus of this study, we decided to further explore this trend. It appeared to be driven mostly by differences between women varying in parenthood status. Childless women were found to report slightly lower germ aversion than women who expected a child, *t* = -2.20, *p* = 0.03 and tended to have slightly lower germ aversion scores than women who had a child, *t* = -1.97, *p* = 0.05 (see Table 1 for all scores). Interestingly, no such differences were observed between groups of men varying in parenthood status. Further, women who had a child reported significantly higher germ aversion than men who had a child, *t* = 3.22, *p* = 0.001. For full results of the ANCOVA analyses as well as results regarding stress level across pregnancy and parenthood see Study 1 supplementary materials (supplementary Tables S1–S3).

**Table 1:** Infectability and germ aversion scores in men and women varying in parenthood status.

Gender	Parenthood status	Mean	SE	95% confidence interval	
				Lower	Upper
Infectability scores					
Woman	Childless	3.25	0.142	2.97	3.53
	Pregnancy	3.27	0.139	3.00	3.54
	Parenthood	3.15	0.124	2.91	3.39
Man	Childless	3.03	0.167	2.70	3.36
	Pregnancy	2.91	0.189	2.54	3.28
	Parenthood	3.05	0.163	2.73	3.38
Germ aversion scores					
Woman	Childless	3.29	0.11	3.07	3.50
	Pregnancy	3.62	0.11	3.41	3.83
	Parenthood	3.58	0.10	3.39	3.77
Man	Childless	3.20	0.13	2.94	3.45
	Pregnancy	3.38	0.15	3.09	3.67
	Parenthood	3.07	0.13	2.82	3.32

## Study 2

To extend and contextualize the findings of Study 1, we decided to explore whether a mother's perceived vulnerability to disease during pregnancy actually predicts her newborn’s health. A sample of pregnant women completed the PVD scale during the second pregnancy trimester and a follow-up survey on their newborn’s health after birth. We expected that mothers with high PVD could give birth to a healthier newborn, possibly due to an increase in prophylactic behaviors limiting risks and pathogens during pregnancy.

### Materials and methods

#### Participants

In Study 2, we invited women during the second pregnancy trimester to complete a health-related survey during pregnancy (T1) and after childbirth (T2). The women were recruited for the project by the experimenters through social media (groups for expectant parents and young mothers), leaflets and posters distributed in medical practices (general practitioners and obstetricians), through facilities organizing educational courses for expectant parents, and using a snowball technique (inviting friends and acquaintances of the participants to the project). All women expected singletons. Out of 221 women who expressed initial interest in the study, 200 completed the T1 survey and the follow-up (T2) measurement. The participants were Polish women aged between 19 and 42 (*M* = 28.84, *SD* = 3.67). One participant was single and 199 participants declared being in a romantic relationship at the time of the study (150 were married and 49 had a partner they cohabited with). One participant reported that her child was born preterm (30^th^ week) and that it was a stillbirth. Her data were not included in the final models. All participants received financial compensation for their participation in the study and provided informed written consent prior to inclusion in the project.

#### Methods

During the pregnancy testing session, participants completed the PVD scale^25^, a demographic survey that included questions on age, height, highest completed education level, self-assessed socio-economic status (SES; rated from 1 indicating much lower than the average to 7, meaning much higher than the average), income per household member (presented as 8 numerical categories of income, with 1 further coded as the lowest and 8 – the highest income category). The mothers also reported body mass before and during pregnancy, partner’s age, height and weight, as well as their general health before the current pregnancy (presence of asthma, allergies, epilepsy, heart condition, diabetes, thyroid gland problems, hypertension, coronary heart disease, anaemia, endometriosis, hormonal issues, other diseases) and pregnancy-related health issues (pregnancy diabetes, thyroid gland problems, hypertension, other diseases; for all information see the study data^33^). Guided by the findings of our Study 1 and the potential impact a mother’s stress has on a child, we also controlled for the mother’s stress at T1 (PSS-10)^32^.

Following childbirth (at T2 meeting), the mothers completed a survey on their newborn using medical records (see *Procedure* for more details). They reported sex, week of childbirth and several variables related to the newborn’s health, namely birth weight (in grams), lowest body mass after birth, day of the lowest body mass, height, head and chest circumference after birth, Apgar scores (scale 1 to 10) assigned to a child by a medical doctor in the 1^st^, 3^rd^, 5^th^ and 10^th^ minute following birth (these were later averaged to compute a mean Apgar score), and general health assessment right after birth described by 12 different indices of a newborn’s health recorded in the child’s documentation (a medical doctor assessed the condition of a head, neck, skin, genitourinary tract, limbs or pelvis, or in newborn’s breathing, heartbeat frequency and sounds, pulse, and overall muscle tone; the doctor would note “1” in a designated space in the health booklet if no abnormalities in a given index were observed). The 12 health indices were further summarized to obtain a general health score, wherein 0 meant the poorest health and 12 indicated the best health. The mothers also reported whether they experienced any problems during childbirth, and whether a child suffered from jaundice.

#### Procedure

The study was part of a larger project on psychological changes experienced by women during pregnancy and after childbirth conducted in 2019. Pregnant mothers completed a survey on several psychological variables (unrelated to the current study). They were invited to contact the experimenter within approximately a month after childbirth to complete a follow-up health-related survey. The majority of the data recorded at the T2 meeting was extracted from the mothers' official medical records and the child's "health booklet." The health booklet is a mandatory instrument in Poland for documenting the health history of pregnant mothers and their children. Parents receive a child's “health booklet” in the hospital where their child is born. It lists information such as the course of pregnancy and birth (including any complications), and the child’s health status after birth (including assessments of 12 aforementioned health indices and the Apgar scores). The mothers brought the medical records and the child's “health booklet” to the T2 meeting and the information were recorded with the assistance of an experimenter. The study was performed in accordance with the Declaration of Helsinki on Biomedical Studies Involving Human Subjects. The procedure was approved by the Ethical Committee of [*University of Wrocław*] and the anonymized data for this study are available on-line^33^.

#### Data analysis

Statistical analyses were performed with SPSS 28.0.1.0 statistical package (IBM) with the level of significance set to α = 0.05. We intended to estimate the predictive value of variables associated with a mother and her health during pregnancy (age, health problems before pregnancy, health problems related to pregnancy, increase in BMI), material situation (income category), stress and PVD subscales on the health outcomes in newborns. Among material situation indices assessed in this study, we also had data regarding self-assessed SES. However, the reported income category and self-assessed socio-economic status (SES) were significantly and positively correlated (*r* = 0.53, *p* < 0.001), and therefore we decided to include just the (more objective) income category to the computed models.

Overall, we computed three separate hierarchical regressions models: (1) Linear regression with a birth weight of a newborn as a dependent variable, (2) Logistic regression with a likelihood to obtain 10 Apgar points (10 Apgar points meaning the best health denoted by 1 vs. any deviations from the score of 10 Apgar points denoted by value 0) as a dependent variable, (3) Logistic regression with likelihood to obtain a maximum value in the newborn general health score as a dependent variable [12-maximum score as indicated by the 12 factors listed above meaning the best health denoted by value 1 vs. any deviations from 12 points score denoted by value 0]. In the two latter cases, we decided to perform a logistic regression analysis predicting any deviations from maximum Apgar or maximum health score, since the data distribution was very skewed both for AGPAR scores and newborn general health scores (skewness of -3.964 and -5.993, respectively).

The first step of each model (Step 1) included the age of a mother, the number of mother’s health problems before pregnancy, the number of mother’s health problems during pregnancy, and the change in the mother’s BMI. The second step of each model (Step 2) involved inclusion of the income category, the subsequent Step 3 additionally comprised stress during pregnancy, while in the fourth step (Step 4) we added perceived infectability and germ aversion PVD scores. The two PVD subscales were positively but weakly correlated with each other, suggesting no multicollinearity problems (which was further confirmed by low values of VIF indices).

### Results

Table 2 presents the descriptive statistics for the analyzed variables. Among newborns born to the mothers included in the project, 155 (77.9%) obtained 10 out of 10 possible mean Apgar points and 162 (81.4%) received the maximum, 12-point health assessment.

**Table 2:** Descriptive data for the mothers included in the study and their newborns.

	Min	Max	*M*	*SE*	*SD*
Mother health problems (0–12)	0.00	5.00	0.70	0.07	0.94
Mother pregnancy health problems (0–4)	0.00	3.00	0.39	0.04	0.60
Corrected change in BMI	-0.10	0.29	0.07	0.00	0.06
Stress (0–40)	1.00	35.00	16.36	0.50	7.10
Average APGAR	3.00	10.00	9.62	0.07	1.02
Perceived Infectability	1.00	7.00	3.13	0.09	1.25
Perceived Germ Aversion	1.25	6.50	3.43	0.07	0.94
Income category (1–8)	1.00	8.00	4.69	0.10	1.45
Self-assessed socio-economic status (1–7)	1.00	7.00	4.76	0.07	1.00
Birthweight (g)	2100.00	5150.00	3433.97	33.82	477.04

The full results of the three regression analyses are presented in Study 2 Supplementary Materials (Tables S4–S6). Neither the linear regression analysis focused on a newborn’s birthweight nor the logistic regression analysis focused on newborn’s maximum health score yielded significant outcomes. In the analysis focused on the maximum Apgar score, the Step 1 regression model including the age of a mother, the mother’s health problems before pregnancy, the mother’s health problems during pregnancy, and change in the mother’s BMI showed that only age significantly predicted the probability of obtaining 10 points on Apgar scale. Younger mothers were more likely to give birth to a child with 10 Apgar points (B = − 0.125, *p* = 0.01). Further, mothers reporting a higher number of health problems before pregnancy tended to be slightly less likely to give birth to a child with 10 Apgar points (B = − 0.345, *p* = 0.085). The logistic regression model was statistically significant, Χ^2^ (4, *N* = 199) = 12.783, *p* = 0.012. The model explained 9.5% (Nagelkerke R^2^) of the variance in maximum Apgar scores and correctly classified 78.4% of cases. Neither income category, stress nor the PVD subscales were found to significantly predict the dependent variables within the computed model and adding these factors in consecutive Steps 2, 3 and 4 did not improve the models’ fit.

## Discussion

Perceived vulnerability to disease is associated with cognitive and behavioral indices of disgust and prophylactic health protection. Here, we found that pregnant women were more likely to exhibit higher levels of PVD as compared with childless peers, although mothers also reported relatively high PVD scores. PVD in men, generally lower than that of the female participants, seemed to be additionally rather independent of their parenthood status. However, as shown by our second study, regardless of the elevated PVD in pregnancy, neither of the perceived vulnerability to disease subscales had a significant impact on the health outcomes of newborns. Birth weight, average Apgar, and general health of a newborn were not associated with the pregnancy-period mother’s PVD score. However, consistent with previous studies indicating that advanced maternal age is associated with an increased risk of adverse outcomes^35,36^, the probability of giving birth to a child with 10 Apgar points was higher in younger mothers. Overall, this research contributes to understanding of the health-oriented beliefs of expecting parents and parents of infants, but it also shows that the possible, PVD-related disease avoidance seems to have a relatively little effect on the basic markers of a newborn’s health.

The results of the first part of our project showed that women, regardless of the parenthood status, perceived their infectability as higher than men, and that the increased female germ aversion tended to interact with the parenthood status. One of the most interesting findings of our study is that slightly elevated germ aversion scores were observed in pregnant women, in line with previous evidence showing an increase of prophylactic behaviors during pregnancy (e.g.,^1,10,11,13^). We may also relate our findings to Behavioral Immune System (BIS) theory, which suggests that human behavior is guided by emotional processes, such as disgust, to avoid pathogen threats, even before they come into contact with the body. These processes result in emotional, behavioral, and cognitive implications, such as sensitivity to disgust and aversion to individuals who pose a risk of pathogen transmission^37^. This relates to a so-called compensatory prophylaxis hypothesis^7^, according to which psychological traits adjust to a current state of immune system, namely that people should be particularly avoidant of pathogen cues when their immune system is compromised. Elevated progesterone level, such as during pregnancy, is related to increased disease avoidance^38^, and our findings show additional support for that assumption. Additionally, general theories of parental investment cost explain that having children is definitely more biologically costly for women than it is for men^39^. This may drive a higher involvement of a mother in prophylactic behaviors and beliefs^22^, although the efficacy of such behaviors may depend on health literacy, an interesting variable that was shown to improve antenatal care and health in pregnancy^40^. Although in Mojoyinola^40^ study health literacy was not significant in terms of pregnancy outcomes, it would nonetheless be an interesting variable to include in further research.

The lower levels of perceived vulnerability to disease in men compared to women was expected^25,41^, but it still seems worthy of consideration that parenthood status had no significant effect on PVD in the male part of our sample. There are few additional explanations for this phenomenon. Extending the previous reasoning, a mother’s infection might have a greater influence on a fetus or even on a child, since mothers usually display more tactile affection in their care of the child^42^. Men may also perceive their role as a parent in different terms than women. Mothers are usually expected to provide comfort and nurturing to a child, while fathers provide play, adventure, and stimulation^43^. This may explain why avoiding infections might be more important for the mothers than for the fathers. Women are also generally more involved in health-related behaviors, such as consulting their symptoms with a doctor, than men^44^. Finally, in line with the “mothers matter more” argument^22^, and the empirical findings reviewed in Sear and Mace^45^, it is mothers and other female relatives who play major role in keeping offspring alive, and fathers’ unconcerned (and seemingly: not malleable) attitudes to disease cues may be a manifestation of that. On one hand, it is possible that Sear and Mace’s conclusion is due to fathers’ insensitivity to disease, as they do not provide protection from germs to their offspring anyway. On the other hand, perhaps it was not evolutionary necessary for them to adjust their psychological traits to parental status, as an offspring was already sufficiently protected by a mother’s psychological disease-related adjustments. These possible explanations require empirical verification, which could include the mentioned perceived role as a parent, as well as participation in childcare, masculinity and femininity, or sociopolitical worldview on gender roles. It is also possible that men are not completely indifferent to vulnerability to disease, but are more concerned with potential health problems of their partner’s rather than of those of their own.

Despite the parenthood-related differences in PVD that we observed in female participants of the Study 1, newborns’ health indices of our participants in Study 2 were not associated with potential, PVD-driven preventive behaviors of the pregnant mothers. Mother's perceived vulnerability to disease during pregnancy may translate more directly into the health of the mother herself, while the health of the babies may be due to a broader range of factors. Provided the immunoregulatory function of the placenta, the fetus is not completely deprived of protection from infections and other external environmental threats that the mother's preventive behaviors would protect it from^4^. Nevertheless, it could be mentioned here that we observed an interesting trend in our analyses: mothers reporting more health issues before pregnancy were slightly less likely to give birth to a child with 10 Apgar points. Although this slight trend needs to be interpreted with caution, it confirms a logical assumption that maternal health is important in the context of pregnancy outcomes. The impact of maternal perceived vulnerability to disease on health (and disease-preventive behaviors) is, however, still rather poorly investigated. Therefore, it seems crucial to further investigate the meaning of maternal elevated perceived vulnerability to disease during pregnancy, provided the apparent health-related psychological changes associated with motherhood. As mentioned previously, pregnant women typically exhibit higher disgust sensitivity compared to others^10,11^. During the COVID-19 pandemic, there was a slight increase in their disgust sensitivity, possibly due to the heightened risk of infection^46^. Additionally, levels of disgust sensitivity appear to fluctuate during pregnancy and post-birth, influenced by women’s illness and even the sex of the fetus^47^. Therefore, these complex interactions and phenomena definitely necessitate further studies.

There are several other health-related behaviors and attitudes which are observed during pregnancy and that influence the infant’s health and pregnancy outcomes, such as prenatal substance use^48^, nutrition^49^ or gestational weight gain^50^. Additionally, pregnant women’s mental state has an effect on the pregnancy and adverse birth outcomes, as shown regarding depression and anxiety^51^. Focusing on predictors included in our research, stress during pregnancy is considered an important factor in infant health and development^29,30^. Exposure to maternal psychological stress during fetal development has been linked to long-term consequences, including neurodevelopmental and respiratory issues, and preterm labor^30,31^. One of the main assumed mechanisms underlying this relationship is the programming effect of prenatal stress on the development of the fetal Hypothalamic—Pituitary—Adrenal axis, leading to alterations in infant stress regulation^52^. However, in our study we observed no apparent parenthood-related stress increase, nor did we find any support for the hypothesis that higher maternal prenatal stress would be associated with adverse neonatal outcomes. A longer longitudinal study could perhaps provide more information on stress effect on the offspring given the solid basis for assuming a significant impact of prenatal maternal stress on later stages of child development^52^ beyond the neonatal health indicators we examined. For example, it was found that maternal mental problems in pregnant women, like anxiety or depression, are associated with problematic behaviors in children after birth^53^. Furthermore, maternal prenatal stress has been shown to affect numerous infant and child outcomes, such as temperament, emotional and stress regulation, cognitive skills and child brain development^54^.

Although our study’s longitudinal design is a definitive strength of this project, future research could benefit from extending follow-up testing to include also older children. Some effects of protective health behaviors in pregnancy, or fetal exposure to stress may not become apparent until later in life. To better understand the links between the elevated PVD and child health, future studies should furthermore examine additional health indicators or involve observations aimed at assessing whether PVD actually translates to increased health protection. Researchers should also consider measuring various indicators of stress, as cortisol levels may better predict adverse infant outcomes resulting from prenatal stress than self-reported measures of stress^55^. Further, we did not collect information regarding sexual orientation in our samples. Although, due to legal restrictions, parenthood remains very infrequent in same-sex relationships in Poland, provided the interesting gender differences we observed, it would be worthy to investigate our predictions in same-sex relationships.

Our study had also some other limitations. Unfortunately, we did not conduct an a priori estimation of the required sample sizes. It resulted in our Study 1 being underpowered (the required sample size estimated post-hoc was 432 individuals, not the 412 we recruited).Since we observed some interesting effects and trends despite this issue, we believe that these results may be particularly meaningful and worthy to investigate further. In Study 2, the sample size was mostly dictated by limitations in resources and logistics—all women were to be interviewed personally by the experimenters both at T1 and T2 and the participants were to receive a financial retribution for their efforts. Nonetheless, despite achieving sufficient power to detect even small effects in that study, we still highlight the need for an a priori estimation of a required sample in further research.

One may also consider whether assessing PVD in the second trimester in Study 2 could have affected our findings. We decided to invite women in this trimester to make the study participation as convenient and comfortable for pregnant participants. However, second trimester of the pregnancy is considered less vulnerable health-wise and psychophysiologically demanding than the first one, with is associated with particularly strong hormonal changes and immunomodulation. In the first trimester, women may also have a greater fear of spontaneous abortion, which could be induced by an environmental threat such as infection. Likewise, in the last trimester, women may also fear premature delivery caused by infections. In that context, our null results can be contrasted with previous findings that women in the first trimester are more disease avoidant^7^. A more recent study reported that disgust sensitivity is particularly high during early pregnancy^56^, although the literature is not fully consistent^47^ with this regard. Another study showed a negative relationship between disgust sensitivity and immune system activity in the first trimester of pregnancy^13^. Moreover, recently it has been shown that sensitivity to disgust is related to sexual steroids’ levels in opposite directions during the first and the third trimester, suggesting that the second trimester may serve as a *plateau* period in this regard^57^. Nevertheless, to gain a better insight to the parenthood-related changes in PVD levels and their potential impact (or lack thereof) on newborns’ health outcomes, future longitudinal projects should ideally include PVD measurements across all three pregnancy trimesters.

## Supplementary Information

Supplementary Tables.

## Data Availability

All data for this study are available at https://osf.io/7fjd6/?view_only=860c131dddc74eaa9889d0a0f985e92c.
