# Supplementary material for: Perceived vulnerability to disease in pregnancy and parenthood and its impact on newborn health
Source: Sci Rep. 2024 Sep 8;14:20907. doi: 10.1038/s41598-024-71870-w (PMC11381513; doi:10.1038/s41598-024-71870-w)
Supplement: Supplementary file 1 — Supplementary Tables. [file 41598_2024_71870_MOESM1_ESM.docx]

**Supplementary Material**

**1. Study 1 Supplementary Materials**

**Table S1.**

Full results of the ANCOVA analysis of the self-assessed infectability

|  | **Sum of Squares** | **df** | **Mean Square** | ***F*** | ***p*** | **η²** |
| --- | --- | --- | --- | --- | --- | --- |
| Overall model | 12.50 | 6 | 2.083 | 1.066 | 0.382 |  |
| Age | 1.91 | 1 | 1.906 | 1.259 | 0.263 | 0.003 |
| Gender_1F2M | 7.82 | 1 | 7.816 | 5.161 | 0.024 | 0.012 |
| STATUS_1Childless_2Pregnant_3Parent | 1.72 | 2 | 0.859 | 0.567 | 0.567 | 0.003 |
| Gender_1F2M ✻ STATUS_1Childless_2Pregnant_3Parent | 1.05 | 2 | 0.527 | 0.348 | 0.706 | 0.002 |
| Residuals | 613.39 | 405 | 1.515 |  |  |  |

**Table S2.**

Full results of the ANCOVA analysis of the self-assessed germ aversion

|  | **Sum of Squares** | **df** | **Mean Square** | ***F*** | ***p*** | **η²** |
| --- | --- | --- | --- | --- | --- | --- |
| Overall model | 16.23 | 6 | 2.705 | 3.67 | 0.001 |  |
| Age | 2.31 | 1 | 2.309 | 2.66 | 0.104 | 0.006 |
| Gender_1F2M | 6.91 | 1 | 6.914 | 7.95 | 0.005 | 0.019 |
| STATUS_1Childless_2Pregnant_3Parent | 3.91 | 2 | 1.953 | 2.25 | 0.107 | 0.011 |
| Gender_1F2M ✻ STATUS_1Childless_2Pregnant_3Parent | 3.10 | 2 | 1.550 | 1.78 | 0.170 | 0.008 |
| Residuals | 352.24 | 405 | 0.870 |  |  |  |

**Table S3.**

Full results of the ANCOVA analysis of the self-assessed stress level

|  | **Sum of Squares** | **df** | **Mean Square** | ***F*** | ***p*** | **η²** |
| --- | --- | --- | --- | --- | --- | --- |
| Overall model | 8.410 | 6 | 1.402 | 3.427 | 0.003 |  |
| Age | <0.001 | 1 | <0.001 | <0.001 | 0.985 | 0.000 |
| Gender_1F2M | 3.076 | 1 | 3.076 | 6.160 | 0.013 | 0.015 |
| STATUS_1Childless_2Pregnant_3Parent | 4.900 | 2 | 2.450 | 4.906 | 0.008 | 0.023 |
| Gender_1F2M ✻ STATUS_1Childless_2Pregnant_3Parent | 0.434 | 2 | 0.217 | 0.435 | 0.648 | 0.002 |
| Residuals | 201.749 | 404 | 0.499 |  |  |  |

For Stress level, the overall interaction effect of gender and parenthood status was non-significant, but the score was significantly affected by gender, *F*(1,404)=6.16, *p*=.013, η²=.015, with women assessing their Stress level as slightly higher than men (*M*=2.84; 95%CI: 2.75-2.93 vs. *M*=2.66; 95%CI: 2.54-2.77). Stress was also tended to be affected by parenthood status, *F*(2,404)=4.91, *p*=.008, η²=.023. Childless participants were found to report slightly higher Stress level than participants who expected a child (*M*=2.90; 95%CI: 2.77-3.03 vs. *M*=2.60; 95%CI: 2.63-2.87, *t*=3.13, *p_Bonferroni_*=.006). The age effect did not reach statistical significance.

Finally, we explored the relationship of the PVD subscales with the Stress level of our participants. Across the whole sample, PVD infectability and germ aversion scores were modestly related to the perceived stress (*r*=.25, *p*<.001 and *r*=.17, *p*<.001, respectively). The correlations were significant for both female (*r*=.21, *p*=.001 and *r*=.11, *p*=.072) and male participants (*r*=.30, *p*<.001 and *r*=.23, *p*<.001).

**2. Study 2 Supplementary Materials**

***Analysis 1. Linear regression – estimating birthweight***

**Table S4.**

Summary of the linear regression model estimating birthweight (model coefficient reported for full model)

|  | **Predictor** | **β stand** | **SE** | ***t*** | ***p*** |
| --- | --- | --- | --- | --- | --- |
| Step 1:  R^2^=-0.02,  *F*(4,194)=0.28, *p*=.893 | Intercept |  | 330.39 | 11.38 | <.001 |
|  | Age of a mother | .01 | 66.35 | .02 | .985 |
|  | Change in BMI | -.05 | 9.75 | -.65 | .519 |
|  | Mother’s health problems before pregnancy | -.02 | 607.25 | -.32 | .749 |
|  | Mother’s health problems during pregnancy | .08 | 42.96 | .95 | .342 |
| Step 2:  ΔR^2^=.001, *p*=.604,  *F*(5,193)=0.28, *p*=.927 | Income level | .03 | 25.18 | .38 | .702 |
| Step 3:  ΔR^2^=.001, *p*=.708,  *F*(6,192)=0.25, *p*=.958 | Stress level | -.03 | 5.03 | -.33 | .744 |
| Step 4:  ΔR^2^=.011, *p*=.360,  *F*(8,190)=0.45, *p*=.892 | Infectability | -.04 | 4.73 | -.54 | .588 |
|  | Germ aversion | -.09 | 4.02 | -1.22 | .224 |

***Analysis 2. Logistic regression – estimating maximum health score***

**Table S5.**

Summary of the logistic regression model estimating maximum health score

|  |  | **Step 1** | | | | **Step 2** | | | | **Step 3** | | | | **Step 4** | | | | |
| --- | --- | --- | --- | --- | --- | --- | --- | --- | --- | --- | --- | --- | --- | --- | --- | --- | --- | --- |
|  | Independent Variable | B | SE | Wald | *p* | B | SE | Wald | *p* | B | SE | Wald | *p* | B | SE | Wald | *p* |  |
| Results of individual predictors | Constant | 1.12 | 1.55 | 0.52 | .471 | 0.96 | 1.56 | 0.38 | .540 | 1.06 | 1.65 | 0.41 | .523 | 0.80 | 1.80 | 0.20 | .659 |  |
|  | Age of a mother | 0.03 | 0.05 | 0.35 | .553 | 0.02 | 0.05 | .019 | .663 | 0.02 | 0.05 | 0.19 | .660 | 0.02 | 0.05 | 0.17 | .677 |  |
|  | Change in BMI | -4.63 | 3.19 | 2.11 | .147 | -4.60 | 3.17 | 2.11 | .146 | -4.56 | 3.17 | 2.07 | .150 | -4.56 | 3.18 | 2.07 | .151 |  |
|  | Mother’s health problems before pregnancy | 0.01 | 0.22 | 0.00 | .985 | .001 | 0.22 | 0.01 | .971 | 0.01 | 0.22 | 0.01 | .964 | -0.01 | 0.22 | 0.01 | .974 |  |
|  | Mother’s health problems during pregnancy | -0.33 | 0.33 | 1.02 | .313 | -0.33 | 0.33 | 0.99 | .319 | -0.33 | 0.33 | 0.97 | .324 | -0.32 | 0.34 | 0.90 | .344 |  |
|  | Income level | - | - | - | - | 0.08 | 0.13 | 0.37 | .544 | 0.07 | 0.14 | 0.29 | .592 | 0.07 | 0.14 | 0.28 | .594 |  |
|  | Stress level | - | - | - | - | - | - | - | - | -0.01 | 0.03 | 0.03 | .855 | -0.01 | 0.03 | 0.04 | .839 |  |
|  | Infectability | - | - | - | - | - | - | - | - | - | - | - | - | 0.01 | 0.02 | 0.02 | .889 |  |
|  | Germ aversion | - | - | - | - | - | - | - | - | - | - | - | - | 0.01 | 0.03 | 0.12 | .731 |  |
| Results of the overall model | -2LLχ2 | 185.556 (df = 4, *p* = .332) | | | | 186.188 (df = 5, *p* = .421) | | | | 186.154 (df = 6, *p* = .545) | | | | 186.000 (df = 8, *p* = .742) | | | | |
|  | R^2^ (Cox & Snell) | .023 | | | | .025 | | | | .025 | | | | .026 | | | | |
|  | R^2^ (Nagelkerke) | .037 | | | | .040 | | | | .040 | | | | .041 | | | | |

***Analysis 3 – Estimating maximum APGAR score***

**Table S6.**

Summary of the logistic regression model estimating maximum APGAR score

|  |  | **Step 1** | | | | **Step 2** | | | | **Step 3** | | | | **Step 4** | | | | |
| --- | --- | --- | --- | --- | --- | --- | --- | --- | --- | --- | --- | --- | --- | --- | --- | --- | --- | --- |
|  | Independent Variable | B | SE | Wald | *p* | B | SE | Wald | *p* | B | SE | Wald | *p* | B | SE | Wald | *p* |  |
| Results of individual predictors | Constant | 5.12 | 1.49 | 11.77 | <.001 | 5.05 | 1.52 | 10.99 | <.001 | 4.98 | 1.58 | 9.90 | .002 | 5.25 | 1.72 | 9.30 | .002 |  |
|  | Age of a mother | -0.13 | 0.05 | 6.64 | .010 | -0.13 | 0.05 | 6.63 | .010 | -0.13 | 0.05 | 6.65 | .010 | -0.12 | 0.05 | 6.27 | .012 |  |
|  | Change in BMI | 1.68 | 3.28 | 0.26 | .610 | 1.61 | 3.28 | 0.24 | .624 | 1.62 | 3.29 | 0.24 | .622 | 1.51 | 3.31 | 0.21 | .648 |  |
|  | Mother’s health problems before pregnancy | -0.35 | 0.20 | 2.97 | .085 | -0.35 | 0.20 | 2.96 | .085 | -0.35 | 0.20 | 2.98 | .084 | -0.34 | 0.21 | 2.67 | .103 |  |
|  | Mother’s health problems during pregnancy | -0.15 | 0.34 | 0.20 | .656 | -0.15 | 0.34 | 0.18 | .668 | -0.15 | 0.34 | 0.19 | .662 | -0.16 | 0.34 | 0.22 | .642 |  |
|  | Income level | - | - | - | - | 0.03 | 0.13 | 0.05 | .823 | 0.03 | 0.13 | 0.07 | .799 | 0.04 | 0.13 | 0.10 | .752 |  |
|  | Stress level | - | - | - | - | - | - | - | - | 0.01 | 0.03 | 0.03 | .873 | 0.01 | 0.03 | 0.05 | .822 |  |
|  | Infectability | - | - | - | - | - | - | - | - | - | - | - | - | 0.01 | 0.02 | 0.13 | .719 |  |
|  | Germ aversion | - | - | - | - | - | - | - | - | - | - | - | - | -0.02 | 0.02 | 0.73 | .394 |  |
| Results of the overall model | -2LLχ2 | 197.48 (df = 4, *p* = .012) | | | | 197.432 (df = 5, *p* = .025) | | | | 197.406 (df = 6, *p* = .045) | | | | 196.629 (df = 8, *p* = .092) | | | | |
|  | R^2^ (Cox & Snell) | .062 | | | | .062 | | | | .063 | | | | .066 | | | | |
|  | R^2^ (Nagelkerke) | .095 | | | | .096 | | | | .096 | | | | .102 | | | | |
